# Supplementary figures and images for: Vibrational stress affects extracellular signal-regulated kinases activation and cytoskeleton structure in human keratinocytes
Source: PLoS One. 2020 Apr 8;15(4):e0231174. doi: 10.1371/journal.pone.0231174 (PMC7141684; doi:10.1371/journal.pone.0231174)

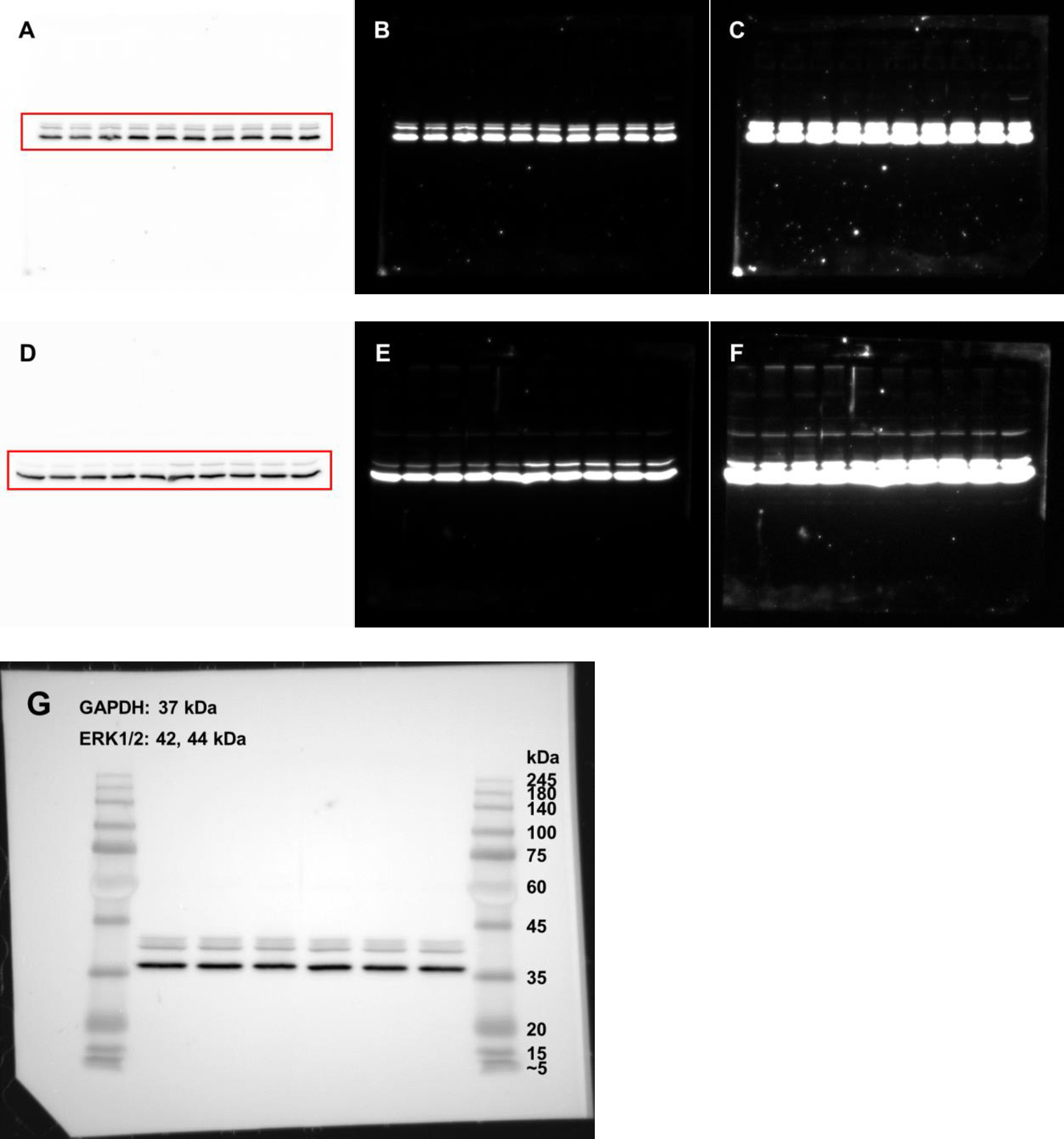

Supplement: S1 Fig — (A) Full length image of total-ERK1/2 and GAPDH; (B), (C) inverted and overexposed version of (A) to confirm the borderline of the polyvinylidene difluoride (PVDF) membrane; (D) full length image of p-ERK1/2 and GAPDH; (E), (F) inverted and overexposed version of (D) to confirm the borderline of the PVDF membrane; (G) western blot image of total-ERK1/2 and GAPDH with size markers. Red lines show the cropping locations. (TIF) [file pone.0231174.s001.tif]
