# Supplementary material for: Vibrational stress affects extracellular signal-regulated kinases activation and cytoskeleton structure in human keratinocytes
Source: PLoS One. 2020 Apr 8;15(4):e0231174. doi: 10.1371/journal.pone.0231174 (PMC7141684; doi:10.1371/journal.pone.0231174)
Supplement: Attachment — Submitted filename: (Plos One) Response to Reviewers Comments 20200216.docx [file pone.0231174.s002.docx]

February 22, 2020

We thank the reviewers for the insightful and constructive suggestions. We are addressing them below. The revised manuscript has been much improved. The reviewers’ comments are in *italic*, while responses are in plain text.

***Reviewer #1’s comments:***

*I have thoroughly reviewed the manuscript entitled "Vibrational stress affects extracellular signal-regulated kinase activation and cytoskeleton structure in human keratinocytes". This manuscript describes the effects of mechanical vibration stress on human keratinocytes using an in vitro vibrational cell culture model. The manuscript is well written, the experiments are clearly presented the reference list is adequate. This research can be promising mechanical energy-based treatment for enhancing wound healing efficiency, as a result I strongly recommend the publication of the manuscript as it is. Additionally, some suggestions were listed as follows for the authors to improving the work.*

*1) This manuscript investigated the effects of mechanical vibration using the in vitro model. Experimental results of this work are interesting, although the innovative points of this paper did not demonstrated clearly in the introduction part, experimental section, and results and discussions is not clearly presented. Thus, I suggest that the authors summarize the specific characteristics and differences of proposed method in the form of a Table by comparing conventional methods.*

**Response:** We thank the reviewer for the comment. As the reviewer suggested, we summarized the characteristics and differences of the current study compared to previous ones in the form of a table.

**Table 2.** Comparison of the findings and limitations of studies related to the wound healing effects of vibration

| Study type | Vibratory condition | Findings | Limitations | Ref. |
| --- | --- | --- | --- | --- |
| *in vivo* | 45 Hz (0.4 *g*) | - Increased angiogenesis and wound healing - Reduced neutrophil accumulation and increased macrophage accumulation - Increased IGF-1, VEGF, MCP-1 expressions | - Did not describe the wound healing mechanism at the cellular level | 15 |
| *in vivo* | 35 Hz (0.3 *g*) | - Increased blood microcirculation - Reduced blood glucose level - Increased GLUT4 expression | - Did not describe the wound healing mechanism at the cellular level | 16 |
| *in vitro* | 60 Hz (0.3 *g*) | - Did not affect cell proliferation | - Focused on osteogenic differentiation - Used rat mesenchymal stromal cells | 30 |
| *in vitro* | 205 Hz (4 *g*) | - Increased metabolic activity (but only slightly) | - Focused on characterization of vocal fold-derived cells - Did not use low-magnitude high-frequency vibratory condition - Used vocal fold-derived cells | 31, 32 |
| *in vitro* | 50 Hz (0.05 *g* to 0.9 *g*) | - Decreased proliferation | - Focused on osteogenic differentiation - Used human periodontal ligament stem cells | 33 |
| *in vitro* | 45 Hz (0.8 *g*) | - Induced cytoskeletal rearrangement - Increased ERK1/2 activation - Increased HB-EGF, EGFR expressions - Increased cell migration (but the increase was not statistically significant) | - Did not demonstrate significant beneficial effects on cell proliferation and migration | This study |
| IGF-1, insulin-like growth factor 1; VEGF, vascular endothelial growth factor; MCP-1, monocyte chemoattractant protein 1; GLUT4, glucose transporter type 4; ERK1/2, extracellular signal-regulated kinases 1 and 2; HB-EGF, heparin-binding epidermal growth factor-like growth factor; EGFR, epidermal growth factor receptor | | | | |

We added it in Discussion section of current manuscript.

*2) The authors should improve their paper-writing with correct grammar and scientific scope.*

**Response:** As the reviewer suggested, we corrected grammar and scientific scope throughout the manuscript.

***Reviewer #2’s comments:***

*Manuscript designed with scientific and technical merits However few minor modifications are required.*

*1) In abstract kindly mention the specific immuno-stains and protein markers to perform the molecular pathway.*

**Response:** We added the specific immunostaining and western blot markers in that sentences for the relevant information in Abstract as the reviewer suggested.

**“ ~** The results of immunostaining (F-actin), western blot (ERK1/2), and RT-qPCR (FGF-2, PDGF-B, HB-EGF, TGF-β1, EGFR, and KGFR) analyses demonstrated that the applied vibration resulted in rearrangement of the cytoskeleton, leading to activation of ERK1/2, one of the MAPK signaling pathways, and upregulation of the gene expression levels of HB-EGF and EGFR.~”

*2) Cross check for mentioning full name followed by abbreviations.*

**Response:** We thank the reviewer for the comment. As the reviewer suggested, we checked abbreviations and their full names, and revised some errors.

*3) Conclusion should cover pros and cons of the outcome of research.*

**Response:** We agreed with reviewer’s comment. As you suggested, we added the related contents in the Discussion section in the form of a table.

*4) Scientific expressions in terms of English should be edited by any science subject experts at professional level.*

**Response:** As you suggested, we revised and improved scientific expressions throughout the manuscript.

*5) Check spell errors throughout the manuscript.*

**Response:** As you suggested, we checked spell errors and revised the manuscript.

We also corrected the reference #30.
